# *In vitro* study of interaction of 17β-hydroxysteroid dehydrogenase type 10 and cyclophilin D and its potential implications for Alzheimer’s disease

**DOI:** 10.1038/s41598-019-53157-7

**Published:** 2019-11-13

**Authors:** Erika Hemmerová, Tomáš Špringer, Zdenka Krištofiková, Jiří Homola

**Affiliations:** 1 Institute of Photonics and Electronics of the Czech Academy of Sciences, Chaberská 57, 182 51 Prague, Czech Republic; 2 National Institute of Mental Health, Topolová 748, 250 67 Klecany, Czech Republic

**Keywords:** Molecular neuroscience, Mechanisms of disease, Molecular medicine, Alzheimer's disease

## Abstract

In early stages of Alzheimer’s disease (AD), amyloid-β (Aβ) accumulates in neuronal mitochondria where it interacts with a number of biomolecules including 17beta-hydroxysteroide dehydrogenase 10 (17β-HSD10) and cyclophilin D (cypD). It has been hypothesized that 17β-HSD10 interacts with cypD preventing it from opening mitochondrial permeability transition pores and that its regulation during AD may be affected by the accumulation of Aβ. In this work, we demonstrate for the first time that 17β-HSD10 and cypD form a stable complex *in vitro*. Furthermore, we show that factors, such as pH, ionic environment and the presence of Aβ, affect the ability of 17β-HSD10 to bind cypD. We demonstrate that K^+^ and Mg^2+^ ions present at low levels may facilitate this binding. We also show that different fragments of Aβ (Aβ_1–40_ and Aβ_1–42_) affect the interaction between 17β-HSD10 and cypD differently and that Aβ_1–42_ (in contrast to Aβ_1–40_) is capable of simultaneously binding both 17β-HSD10 and cypD in a tri-complex.

## Introduction

Research into interactions between biomolecules represents an important route to the understanding of life at a molecular and cellular level. Expanding the knowledge of molecular processes associated with an onset and progression of a disease is a major challenge for modern science with potential significant implications for the development of new diagnostic and treatment modalities. Surface plasmon resonance (SPR) biosensors are an essential technology for the real-time label-free investigation of biomolecular interactions^1^. In recent years, SPR biosensors have been applied for the study of interactions of a variety of biomolecules (e.g., proteins, nucleic acids, and lipids), providing new insights into equilibrium and kinetic aspects of biomolecular interactions and relationships between interacting biomolecules (e.g., binding stoichiometry, epitope mapping)^2,3^. In this work, we use SPR biosensor technology to investigate interactions of proteins implicated in Alzheimer’s disease (AD).

AD is a chronic neurodegenerative disease that is characterized by the progressive decline of memory and cognitive functions due to extensive neuronal death, and despite decades of intensive research, the progression of the disease is still not fully understood and therefore no effective cure is yet available. One of the main pathological hallmarks found in the affected parts of brains of AD patients are senile plaques formed by the aggregates of extracellular amyloid-β peptide (Aβ). Aβ is a peptide released at different sizes, of which 40aa long residues (Aβ_1–40_) represent ~80–90% of the physiologically secreted Aβ fragments, followed by 42aa long residues (Aβ_1–42_) representing ~5–10% of total Aβ fragments^4^. During AD, mutations in the cleavage pathway can lead to an increased production of Aβ and/or preferential production of Aβ_1–42_ over Aβ_1–40_ leading to oligomerization of Aβ and formation of aggregates^5–7^. As Aβ_1–42_ is more prone to the oligomerization than Aβ_1–40_^8,9^, it plays a crucial pathological role in AD. Recent studies suggest that the role of Aβ in AD may be more complex than just the formation of plaques^10,11^. It has been highlighted that in early stages of AD, Aβ enters neuronal cells and accumulates, among other places, in the synaptic mitochondria^12,13^. Inside mitochondria, it binds other biomolecules of mitochondrial matrix, such as 17beta−hydroxysteroid dehydrogenase type 10 (17β-HSD10)^14–16^ or cyclophilin D (cypD)^17,18^ and thereby contributes to the progression of AD.

17β-HSD10 is a protein which is involved in mitochondrial metabolism^19^ and in the maintenance of mitochondrial integrity under metabolic stress^20^. The interaction between 17β-HSD10 and monomeric Aβ has very little effect on the enzymatic function of 17β-HSD10^14^; however, the oligomeric Aβ inhibits its activity as it changes the conformation of 17β-HSD10 preventing it from binding its cofactor NAD^+ ^^21^. CypD is also located in the mitochondrial matrix and due to oxidative and other cellular stresses may be translocated to the inner mitochondrial membrane. This leads to the formation and opening of mitochondrial permeability transition pores (mPTP), resulting in the collapse of membrane potential and activation of apoptotic cell mechanisms^18,22^. Both fragments of Aβ (Aβ_1–40_ and Aβ_1–42_) interact with  cypD; Aβ_1–42_ exhibiting higher affinity to cypD than Aβ_1–40_ and the oligomeric form exhibiting higher affinity to cypD than the monomeric form^17,18^.

Interactions of Aβ with both 17β-HSD10 and cypD were linked with pathological processes, such as disturbed mitochondrial energy metabolism, Ca^2+^ homeostasis, membrane potential change, generation of reactive oxygen species (ROS) and other mitochondrial dysfunctions^23,24^. In addition, it was hypothesized that 17β-HSD10 and cypD form a complex and that excessive accumulation of Aβ during AD may disrupt this complex resulting in the upregulation of free cypD, increased mPTP opening and pronounced apoptosis. However, this hypothesis was based on studies using indirect methods, such as fluorescence co-localization or immunoprecipitation^14,25^, and no direct proof of the binding between 17β-HSD10 and cypD has been provided yet.

**Figure 1:**
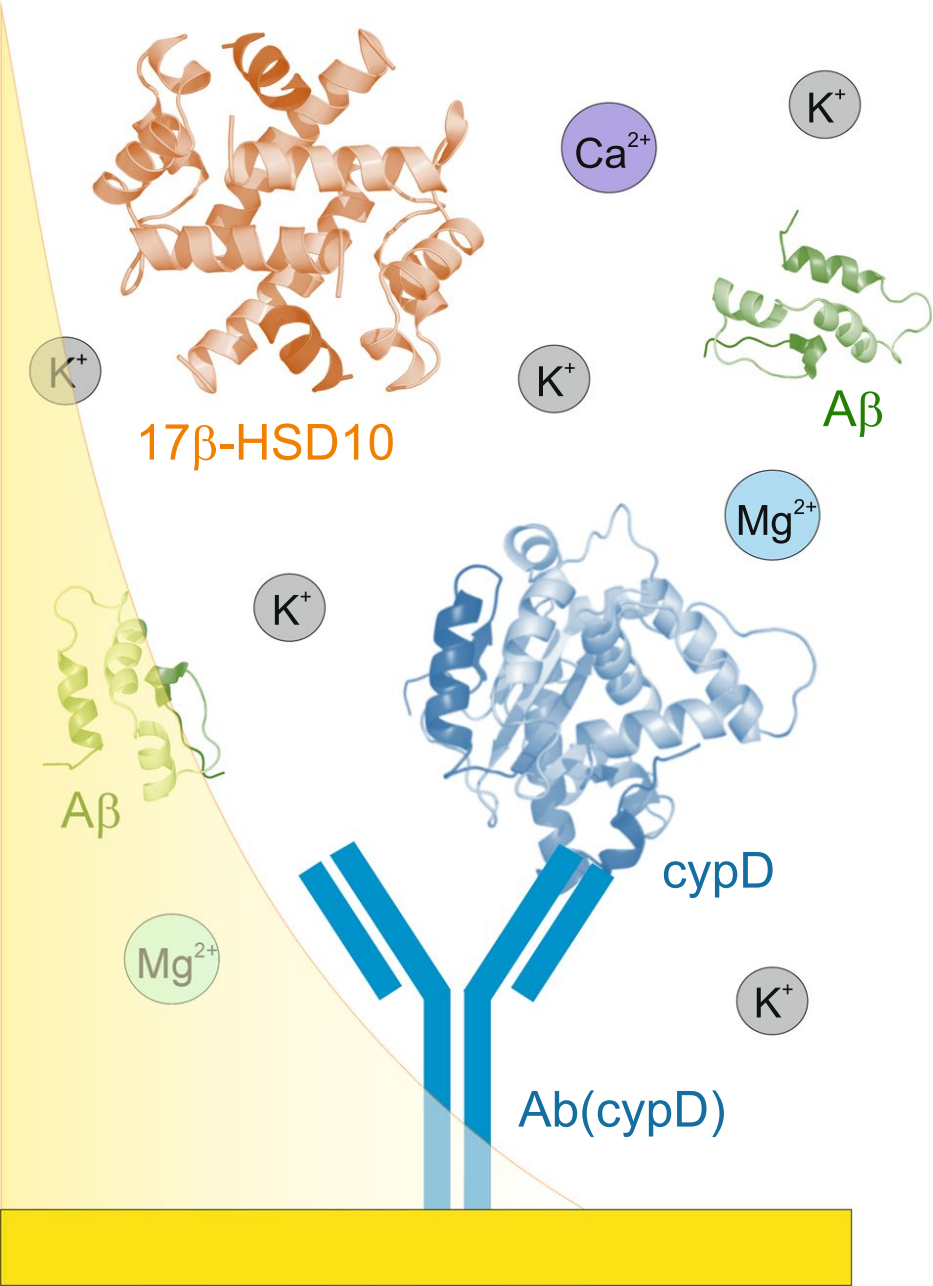
Design of the experimental layout for investigation of the interaction between 17β-HSD10 and cypD using an SPR biosensor.

In this work, we use the SPR biosensor method to study the interaction between 17β-HSD10 and cypD in a direct manner and under variable physiologically relevant environmental conditions (Fig. 1). As the concentration of ions in the mitochondrial matrix fluctuates in response to the perturbations in the cytosolic environment as well as to the temporal metabolic processes^26,27^, we study the effect of ions on this interaction. In particular, we investigate how the binding between 17β-HSD10 and cypD is influenced by concentrations of the most relevant ions, such as K^+^, Mg^2+^, and Ca^2+^. Finally, in order to explore the hypothesis proposed by Yan and Stern, we evaluate the effect of the presence of two different Aβ fragments, Aβ_1–40_ and Aβ_1–42_ (exhibiting different oligomerization dispositions) on the interaction between 17β-HSD10 and cypD to enable deeper understanding of processes taking places during the early stages of AD.

## Results

### Study of interaction between 17β-HSD10 and cypD under variable environmental conditions

In this part, we present the results of our study of the interaction between 17β-HSD10 and cypD under different environmental conditions, such as pH and concentrations of ions. As physiological values of pH in the mitochondrial matrix are believed to be 7.8^28^ or lower (down to 7.0)^29^, we considered two pH values - 7.4 and 7.8. The concentration range for K^+^ and Mg^2+^ (the most abundant mono- and bi-valent ions present in the mitochondrial matrix) was selected to span from 0 mM to the physiological concentrations reported for the mitochondrial matrix: 140 mM for K^+ ^^30^ and 2.5 mM for Mg^2+ ^^31^. While the binding experiments were performed at a temperature of 25 °C for all the selected combinations of environmental parameters to facilitate comparison with the previous studies, the selected experiments were repeated at a physiologically more relevant temperature of 37 °C to assess the effect of temperature.

**Figure 2:**
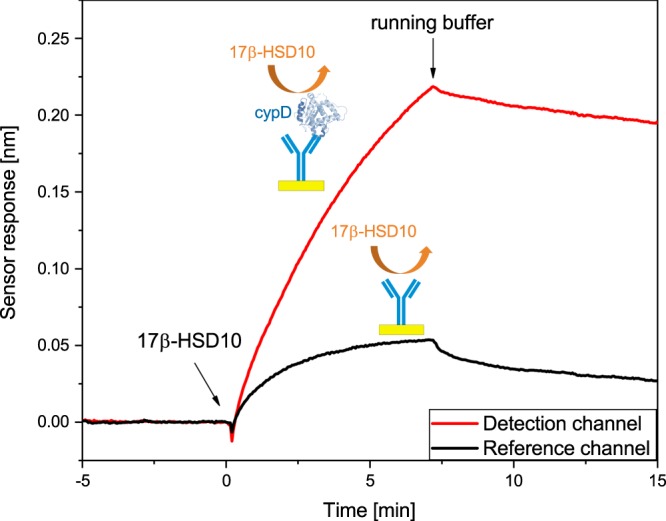
Sensorgram corresponding to the binding of 17β-HSD10 to the surface with and without immobilized cypD.

Figure 2 shows the sensorgram corresponding to the binding of 17β-HSD10 to the surface of the sensor with and without immobilized cypD. A much higher sensor response can be seen in the sensor channel with immobilized cypD, which indicates that 17β-HSD10 recognizes cypD and is able to bind to it. After the supply of 17β-HSD10 is interrupted (t = 7 minutes), the sensor response in the sensor channel with the immobilized cypD decreases only slightly which implies that the complex formed by 17β-HSD10 and cypD is stable.

**Figure 3:**
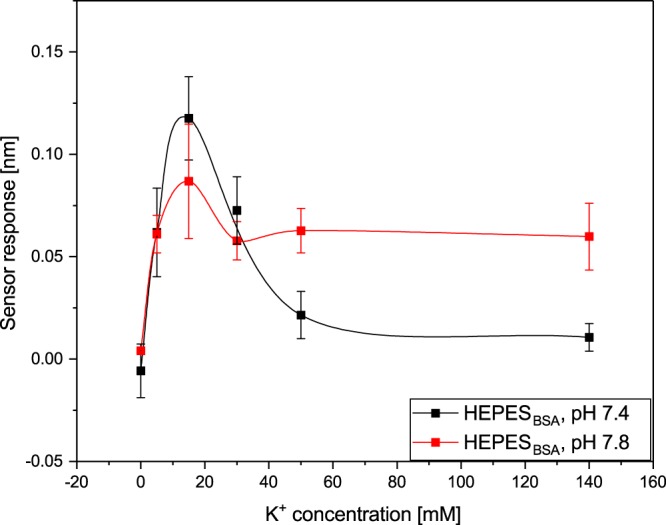
Dependence of the binding between 17β-HSD10 and cypD on concentration of K^+^ obtained at a pH of 7.4 and 7.8.

**Figure 4:**
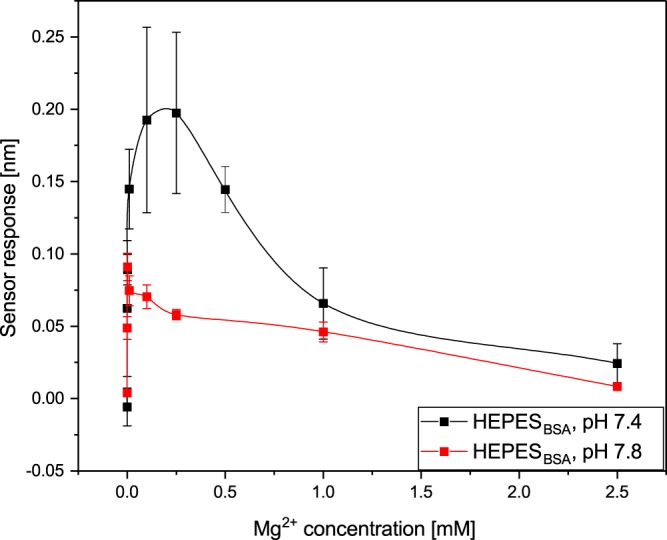
Dependence of the binding between 17β-HSD10 and cypD on concentration of Mg^2+^ obtained at a pH of 7.4 and 7.8.

Figures 3 and 4 show the dependence of the sensor response (corresponding to the binding of 17β-HSD10 to the immobilized cypD) on the concentration of K^+^ and Mg^2+^ measured at two different pH values. For all the types of ions and pH values, the data indicate that the efficiency of the binding between 17β-HSD10 and cypD approaches zero in the absence of ions and increases with an increasing concentration of ions, until it reaches a maximum value. A further increase in the concentration of ions results in a decrease in the binding efficiency. However, whereas the concentration of K^+^ corresponding to the maximum binding efficiency is about the same (15 mM) for both pH values, the maximum binding efficiency in the presence of Mg^2+^ ions occurs at 1 μM (pH 7.8) and 0.25 mM (pH 7.4). In addition, the binding is more pronounced in the presence of Mg^2+^ than in the presence of K^+^ (for both the pH values, albeit only slightly at pH 7.8) and at a pH of 7.4 than at a pH of 7.8 (for both types of ions). Therefore, a pH of 7.4 was selected for use in further studies.

**Figure 5:**
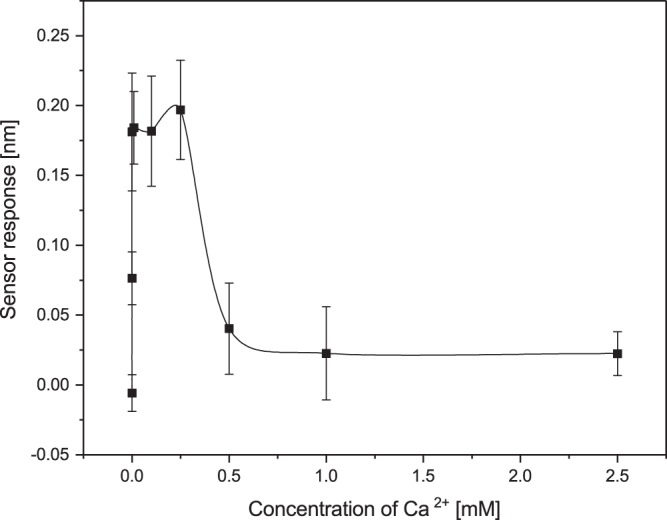
Dependence of the binding between 17β-HSD10 and cypD on concentration of Ca^2+^ obtained at a pH of 7.4.

In order to further characterize the effect of ions on the interaction between 17β-HSD10 and cypD, we studied the effect of Ca^2+^ ions that play a prominent role in mitochondrial metabolism^32^ and apoptosis^33^. As follows from Fig. 5, the binding efficiency between 17β-HSD10 and cypD in the presence of Ca^2+^ follows the same trend as the binding efficiency in the presence of the other two ions and the maximum binding efficiency takes place at a concentration of Ca^2+^ of 0.25 mM.

**Figure 6:**
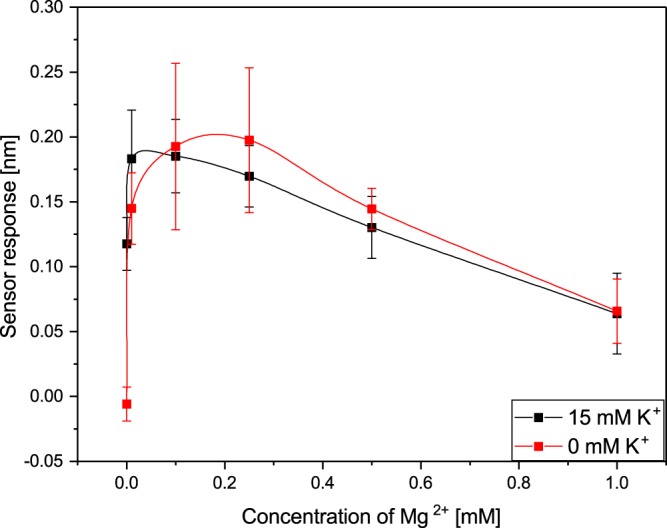
Dependence of the binding of 17β-HSD10 to cypD on concentration of Mg^2+^ in the presence and absence of 15 mM K^+^ obtained at a pH of 7.4.

**Figure 7:**
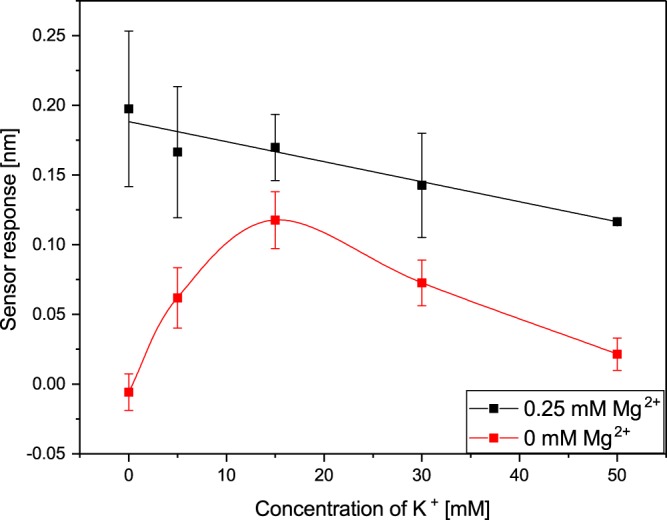
Dependence of the binding of 17β-HSD10 to cypD on concentration of K^+^ in the presence and absence of 0.25 mM Mg^2+^ obtained at a pH of 7.4.

The mixture of ions inside the mitochondrial matrix may collectively affect biomolecular interactions in mitochondria. Therefore, besides the investigation of the effect of individual ions on the binding between 17β-HSD10 and cypD, we also studied the effect of simultaneously present K^+^ and Mg^2+^ ions. In this study, we kept the concentration of one ion constant (at the concentration for which the binding efficiency was the highest) and varied the concentration of the other. The corresponding results in Figs 6 and 7 illustrate how the presence of K^+^ and Mg^2+^ influences the binding between 17β-HSD10 and cypD.

The results depicted in Fig. 6 indicate that at low Mg^2+^ concentrations, the presence of 15 mM K^+^ increases the binding efficiency, whereas at higher Mg^2+^ concentrations, the binding efficiency in the presence of both K^+^ and Mg^2+^ is comparable with that measured in the presence of Mg^2+^ only. Furthermore, Fig. 7 suggests that at low K^+^ concentrations, the presence of 0.25 mM Mg^2+^ increased the efficiency of binding between 17β-HSD10 and cypD. A further increase in K^+^ concentration resulted in a decrease in the binding efficiency for both the combined K^+^ and Mg^2+^ and individual K^+^. The binding efficiency was approximately twice as high for combined Mg^2+^ and K^+^ in comparison with that measured in the presence of K^+^ only.

Based on these experiments, HEPES with 15 mM K^+^ and 0.1 mM Mg^2+^ was selected to be used in further experiments as these conditions appear to favour binding between 17β-HSD10 and cypD.

As follows from Supplementary Fig. S2 and Supplementary Table S3 (see Supplementary Information), 17β-HSD10 and cypD form a complex both at 37 °C and 25 °C. In addition, the interactions of 17β-HSD10 and cypD realized at the two different temperatures generated only slightly different sensor responses (within the error of the measurement).

### Study of interaction between 17β-HSD10 and cypD in the presence of Aβ

**Figure 8:**
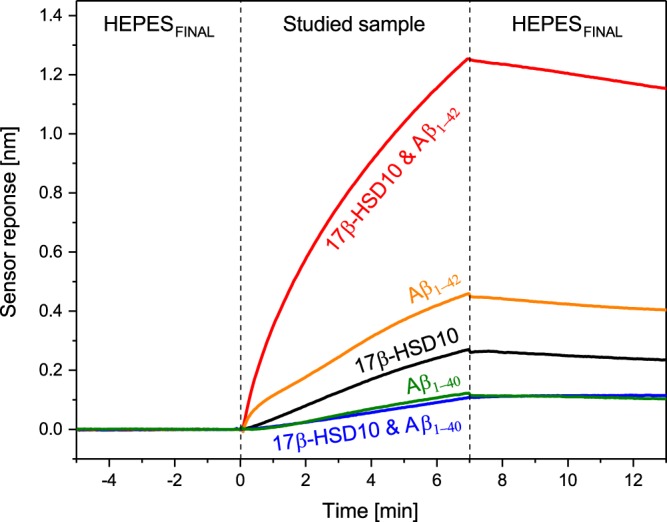
Sensorgram corresponding to the binding of 17β-HSD10 incubated with Aβ (Aβ_1–40_ or Aβ_1–42_), and the binding of individual 17β-HSD10, Aβ_1–42_ and Aβ_1–40_ to the immobilized cypD.

We evaluated whether and how the presence of Aβ_1–40_ or Aβ_1–42_ influences the interaction between 17β-HSD10 and cypD. Figure 8 shows sensorgrams corresponding to the binding of 17β-HSD10 incubated with Aβ (Aβ_1–40_ or Aβ_1–42_), and the binding of individual 17β-HSD10, Aβ_1–40_ and Aβ_1–42_ to cypD immobilized on the surface of an SPR chip. There is a substantial difference in the effect of the two different types of Aβ. Aβ_1–40_ incubated with 17β-HSD10 generates a sensor response that is lower than individual 17β-HSD10 and comparable to that of individual Aβ_1–40_. In contrast, Aβ_1–42_ incubated with 17β-HSD10 generates a sensor response that is higher than the sensor responses corresponding to the individual binding partners (17β-HSD10 or Aβ_1–42_), and is even higher than the two added together.

The results of the interaction study obtained with one of the interacting partners (cypD) immobilized on the surface of an SPR sensor were compared to those obtained with all interacting partners present in solution. In this experimental format, cypD, 17β-HSD10 and Aβ (Aβ_1–40_ or Aβ_1–42_) were incubated in solution and flowed along Ab(cypD) that was immobilized on the surface of an SPR chip. Subsequently, the chip was exposed to Ab(17β-HSD10) to confirm the capture of 17β-HSD10. Supplementary Fig. S3 (see Supplementary Information) shows the binding of Ab(17β-HSD10) to the sensor surface that was previously exposed to the mixture of 17β-HSD10 and cypD, which confirms that the 17β-HSD10/cypD complex was formed. 17β-HSD10 and cypD incubated with Aβ_1–42_ generates a sensor response to Ab(17β-HSD10) that is about 6 times higher than 17β-HSD10 and cypD incubated in the absence of Aβ_1–42_; the incubation with Aβ_1–40_ reduces the sensor response to Ab(17β-HSD10) by about one half. The binding of Ab(17β-HSD10) to the sensor surface exposed to reference solutions containing different combinations of the interacting molecules (Solutions 4–12 in Supplementary Table S2) produces sensor responses significantly smaller than those obtained when using solutions of cypD, 17β-HSD10 and Aβ (see Supplementary Fig. S4). This confirms that the binding was specific.

The data collected in both experimental formats indicates that: 1) Aβ_1–40_ as well as Aβ_1–42_ bind both 17β-HSD10 and cypD and 2) Aβ_1–42_ forms a complex with 17β-HSD10 and cypD containing all three binding partners (a tri-complex), whereas Aβ_1–40_ is not able to interact simultaneously with both proteins. This implies that the binding of free Aβ_1–40_ that is present in the sample in excess, would be responsible for the majority of the sensor response to the binding of the mixture of 17β-HSD10 with Aβ_1–40_ shown in Fig. 8.

**Figure 9:**
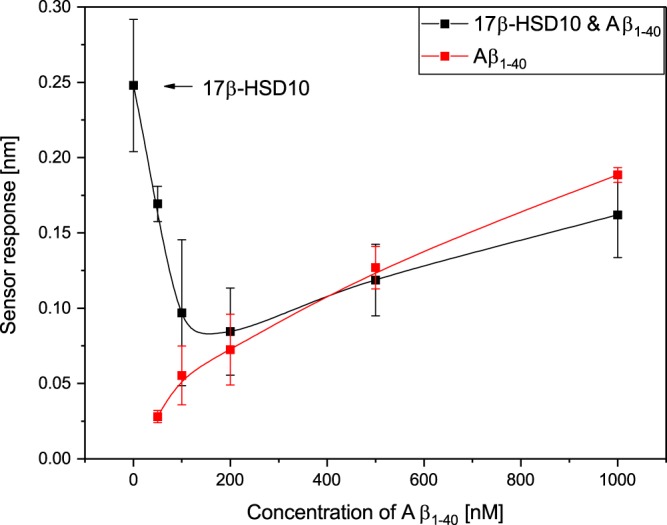
Dependence of the binding of Aβ_1–40_ and Aβ_1–40_ incubated with17β-HSD10 to the immobilized cypD on concentration of Ab_1–40_.

In order to confirm this hypothesis and to correlate the sensor response generated by the binding of Aβ_1–40_ incubated with 17β-HSD10 to the excess of Aβ_1–40_ in the mixture, the sensor response to mixtures with different concentrations of Aβ_1–40_ was measured (molar ratio of Aβ_1–40_ to 17β-HSD10 ranging from 1:2 to 10:1, with the concentration of 17β-HSD10 kept constant). As depicted in Fig. 9, the sensor response to individual Aβ_1–40_ increased with an increasing concentration of Aβ_1–40_. Conversely, the sensor response to Aβ_1–40_ incubated with 17β-HSD10 decreased with an increasing concentration of Aβ_1–40_ until it became comparable with the sensor response corresponding to the binding of individual Aβ_1–40_ (around a concentration of Aβ_1–40_ of 200 nM) and then it started to grow following the same trend as that observed for the binding of individual Aβ_1–40_. This suggests that Aβ_1–40_ binds 17β-HSD10 and the formed complex is not able to bind to cypD. Therefore, at low concentrations of Aβ_1–40_, the sensor response is predominantly caused by the binding of 17β-HSD10, whereas at high concentrations of Aβ_1–40_, it is caused mainly by the binding of free Aβ_1–40_. Results of analogous experiments performed with a variable concentration of Aβ_1–42_ are shown in Supplementary Fig. S5 (see Supplementary Information). Figure S5 shows that the sensor response to the binding of Aβ_1–42_ to cypD immobilized on the sensor surface increases linearly with an increasing concentration Aβ_1–42_. The sensor response to the binding of Aβ_1–42_ incubated with 17β-HSD10 to the immobilized cypD exhibits a more complex behavior: while the sensor response is increasing with the concentration of Aβ_1–42_ in general, there is a pronounced local maximum at a concentration of ~100 nM. While our experiments strongly support this deviation from a monotonous trend of the sensor response (it was confirmed by five independent experiments), we do not have plausible explanation of the mechanisms behind this effect. However, within the considered range of concentrations of Aβ_1–42_, the sensor response to the binding of Aβ_1–42_ incubated with 17β-HSD10 was consistently higher than that corresponding to individual Aβ_1–42_, which confirms the formation of a tri-complex.

**Figure 10:**
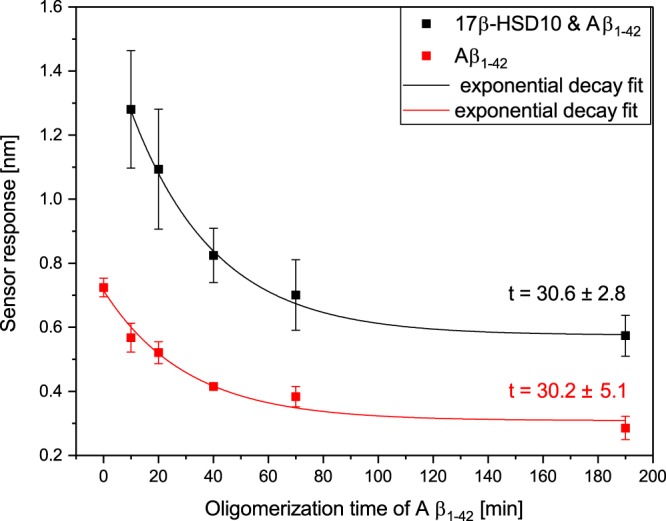
Dependence of the binding of Aβ_1–42_ and 17β-HSD10/Aβ_1–42_ to the immobilized cypD on oligomerization time of Aβ_1–42_.

In order to better understand the mechanism through which Aβ affects the interaction between 17β-HSD10 and cypD, we studied the effect of the degree of oligomerization of Aβ. In this experiment, Aβ in solution was allowed to form oligomers for different periods of time (“oligomerization time”), and then 17β-HSD10 was added into the mixture and the mixture was injected in the SPR sensor. As follows from Fig. 10, Aβ_1–42_ incubated with 17β-HSD10 generates a sensor response about twice as high as individual Aβ_1–42_. This suggests that the 17β-HSD10/Aβ_1–42_ complex binds to the immobilized cypD, which is in agreement with the data presented in Fig. 8. With increasing Aβ_1–42_ oligomerization time, the binding efficiency of both Aβ_1–42_ as well as 17β-HSD10/Aβ_1–42_ to the immobilized cypD decreases. The fitting of the obtained data with the use of a simple exponential decay function yielded time decay constants of about 30 minutes for both the 17β-HSD10/Aβ_1–42_ complex and individual Aβ_1–42_. For comparison, we carried the same experiments with Aβ_1–40_ and did not observe any dependence of the sensor response on the Aβ_1–40_ oligomerization time. This indicates that its oligomerization requires a considerably longer time under the conditions used, which is consistent with the published data^9,34^.

## Discussion

In the first part of our study, we have demonstrated that the two mitochondrial proteins, 17β-HSD10 and cypD, interact with each other forming a stable complex. This is the first report that provides evidence of this interaction and supports the hypothesis of Yan and Stern^14^. Furthermore, we have shown (Figs 3–7) that the interaction between 17β-HSD10 and cypD is significantly affected by environmental conditions such as the pH and concentrations of K^+^, Mg^2+^ or Ca^2+^. We observed considerably higher binding efficiencies for K^+^, Mg^2+^ or Ca^2+^ present at concentrations within a relatively narrow range and at a lower pH value. Furthermore, our data indicate that the effect of bivalent Ca^2+^ and Mg^2+^ ions was rather similar and much stronger than that of monovalent K^+^. This implies that the interaction is not only sensitive to the ionic strength of the environment, but also (at least partially) to the valence of the present ions.

We observed the highest efficiency of the binding between 17β-HSD10 and cypD for a pH of 7.4, and at a concentration of ions around 15 mM of K^+^ and 0.1 mM of Mg^2+^. These values are somewhat lower than the prevalent physiological values in the mitochondrial matrix reported in the literature. However, it should be noted that the mitochondrion is a complex organelle in which concentrations of ions fluctuate in response to metabolic processes and perturbations of the cytosolic environment^26,27^. The data reported in the literature may also be affected by the methodology used. For example, a commonly assumed pH value in mitochondria of 7.8^26,28,30^ originates mostly from studies in which mitochondria were maintained in non-physiological buffers^29^. When mitochondria were maintained under more realistic physiological conditions, considerably lower pH values were observed (down to 7.0^29^). K^+^ is commonly assumed to occur in the mitochondria at concentrations around 140 mM^30,35^; however, much lower K^+^ concentrations (down to 15 mM) have also been observed^36,37^. Mg^2+^ concentrations in the mitochondrial matrix were determined to fall between 0.5 mM and 2.5 mM^38,39^. Therefore, the conditions under which we observed the most efficient binding of 17β-HSD10 to cypD, may be considered physiological. This suggests that our findings may be applicable to interactions in mitochondria and that indeed 17β-HSD10 is able to regulate cypD in mitochondrial matrix. Subsequently, changes in the ionic balance in mitochondrial matrix may disrupt 17β-HSD10/cypD binding and cause the release of cypD, which may result in the opening of mPTP and trigger apoptotic processes. This is a particularly relevant finding, as an excessive accumulation of Ca^2+^ is known to affect membrane potential through the formation and opening of mPTP^33^. We hypothesize that the process of disruption of membrane potential at high Ca^2+^ concentrations is caused by the Ca^2+^-induced disruption of the 17β-HSD10/cypD complex and dysregulation of free cypD.

Similarly to the majority of previous *in vitro* studies investigating the interaction between mitochondrial biomolecules (including the study of interaction between cypD and Aβ^17^ or interaction between 17β-HSD10 and Aβ^21^), we performed the binding experiments predominantly at a temperature of 25 °C. However, comparative experiments carried out under selected environmental conditions at an elevated temperature of 37 °C (temperature of living mitochondria) showed that the binding between 17β-HSD10 and cypD occurs at both the temperatures and that a change in the temperature of 12 °C does not have substantial effect on the interaction.

In the second part of study, we have shown that Aβ affects the binding between 17β-HSD10 and cypD and that the two different fragments of Aβ (Aβ_1–40_ and Aβ_1–42_) influence the binding in a different manner (Fig. 8). Whereas Aβ_1–42_ facilitates the binding between 17β-HSD10 and cypD, Aβ_1–40_ seems to suppress it. We believe that this difference can be explained by the different ability of Aβ_1–40_ and Aβ_1–42_ to form oligomers (oligomerization of Aβ_1–42_ proceeds much faster than that of Aβ_1–40_)^8,9^. The oligomers bind to 17β-HSD10 and the resulting complexes further bind to the immobilized cypD forming a tri-complex, whereas the monomeric Aβ is not able to bind to the two proteins simultaneously. However, with progressing oligomerization of Aβ, its ability to bind cypD decreases (Fig. 10). Interestingly, the ability to bind cypD decreases with the same time constant for Aβ_1–42_ as for 17β-HSD10/Aβ_1–42_. This suggests that the degree of oligomerization of Aβ_1–42_ affects both the binding events (binding of individual Aβ_1–42_ and binding of 17β-HSD10/Aβ_1–42_) in the same fashion. This may be explained by the assumption that both these binding events are driven by the same interaction. Therefore, we hypothesize that the binding of 17β-HSD10/Aβ_1–42_ complex to cypD takes place through Aβ_1–42_.

Our results support the hypothesis by Stern and Yan^14^ who postulated that the presence of Aβ affects the ability of 17β-HSD10 to regulate cypD. However, in contrast to Stern and Yan who suggested that the 17β-HSD10/cypD complex may dissociate in the presence of Aβ, we show that a tri-complex consisting of 17β-HSD10, cypD and Aβ_1–42_ is formed. We suggest that each Aβ form participates in the dysregulation of cypD differently. At physiological concentrations, Aβ_1–40_ remains monomeric and binds 17β-HSD10, thus inhibiting its regulation of free cypD. During AD, Aβ (both fragments Aβ_1–40_ and Aβ_1–42_) accumulates in the mitochondrial matrix, resulting in an increased binding of Aβ_1–40_ to 17β-HSD10 and consequently in an increased level of free cypD triggering the apoptotic processes. Aβ_1–42_ forms a tri-complex with both proteins, thus preventing cypD from translocating to the inner membrane. Excessive oligomerization of Aβ_1–42_ related to AD, suppresses the ability of the 17β-HSD10/Aβ_1–42_ complex to bind cypD, which leads to upregulation of cypD and apoptosis (similarly to Aβ_1–40_). However, it should be noted that at high concentrations Aβ_1–40_ also form oligomers^8,9^ and thus we expect that with progressing AD, the properties of Aβ_1–40_ may approach those of Aβ_1–42_.

## Conclusions

In this study, we show, for the first time, that two proteins related to pathogenesis of Alzheimer’s disease, 17β-HSD10 and cypD, interact and  form a stable complex. The study was performed *in vitro* using the SPR biosensor method; however, we set the experimental conditions in such a way that their key relevant characteristics approached those in the mitochondrial matrix. We have also shown that the interaction between 17β-HSD10 and cypD is sensitive to the ionic composition of the environment. This suggest that changes in the ionic composition which take place in the mitochondrial matrix can impair the regulation of cypD by 17β-HSD10 and lead to apoptosis. In addition, we have demonstrated that the presence of Aβ affects the binding between 17β-HSD10 and cypD and that different fagments of Aβ influence the binding through different mechanisms. While monomeric Aβ can only bind the two proteins separately, oligomeric Aβ can form a tri-complex with 17β-HSD10 and cypD. Increased concentrations and the degree of oligomerization of Aβ during Alzheimer’s disease may hamper the interaction between 17β-HSD10 and cypD, which may result in the dysregulation of cypD by 17β-HSD10 and apoptosis *via* increased opening of mitochondrial permeability transition pores.

## Methods

### Reagents

NaCl, NaOH, KCl, MgCl_2_, CaCl_2_, bovine serum albumin (BSA) and all buffers: sodium acetate (SA10; 10 mM, pH 5.0), MES (10 mM, pH 5.0), HEPES (10 mM), high ionic strength phosphate-buffered saline (PBS_Na_; 10 mM phosphate, 2.9 mM KCl, 750 mM NaCl, pH 7.4), were purchased from Sigma-Aldrich, Czech Republic. Oligo-ethylene glycol thiols 11-mercapto-hexa(ethyleneglycol)undecyloxy acetic acid (HS-C_11_-(EG)_6_-OCH_2_-COOH) and 11-Mercapto-tetra(ethyleneglycol)undecanol (HS-C_11_-(EG)_4_-OH) were purchased from Prochimia, Poland. Ethanolamine hydrochloride (EA), 1-ethyl-3-(3-dimethylaminopropyl)-carbodiimide hydrochloride (EDC) and *N*-hydroxysuccinimide (NHS) were purchased from Biacore, Sweden. All buffers were prepared using deionized Milli-Q water (Merck, Czech Republic). 17β-HSD10 (human, recombinant), cypD (human, recombinant) and an antibody against cypD (Ab(cypD)) were purchased from Fitzgerald, USA, and an antibody against 17β-HSD10 (Ab(17β-HSD10)) from Biolegend, USA. Aβ_1–40_ and Aβ_1–42_ (human, synthetic) were obtained from AnaSpec, USA.

### Surface plasmon resonance (SPR) biosensor

We used a six-channel laboratory SPR biosensor platform based on the prism coupling and wavelength spectroscopy of surface plasmons (Plasmon VI) interfaced with a dispersionless microfluidic system, both developed at the Institute of Photonics and Electronics, Prague^40^. In this platform, the angle of incidence of the light beam is fixed and changes in the excitation (resonance) wavelength of surface plasmons are measured by analysing the spectrum of polychromatic light reflected from an SPR chip attached to the prism coupler. The resonance wavelength is sensitive to changes in the refractive index caused by the binding of molecules to the surface of an SPR chip. In the used platform (resonance wavelength of 750 nm), a shift of 1 nm in the SPR wavelength represents a change in the protein surface coverage of 17 ng/cm^2^. The SPR chips used in this study were prepared by coating microscope glass slides (Marienfeld, Germany) with thin layers of titanium (1–2 nm) and gold (48 nm) prepared via e-beam evaporation in vacuum. The SPR platform was equipped with a temperature stabilization module capable of maintaining a temperature within the microfluidic flow cell with a precision of 0.01 °C. All experiments were performed at a temperature of 25 °C and a flow rate of 20 µl/min unless explicitly stated otherwise.

Prior to the experiments, the surface of an SPR chip was modified by a self-assembled monolayer of mixed thiols, on which Ab(cypD) was immobilized using the amino-coupling method as described previously^41^. Briefly, a clean SPR chip was immersed in a 3:7 molar mixture of HS-C_11_-(EG)_6_-OCH_2_-COOH and HS-C_11_-(EG)_4_-OH (ethanol solution, total concentration of 0.2 mM), then incubated for 10 minutes at 40 °C and then for at least 12 h at room temperature in the dark. Prior to use, the chip was rinsed with ethanol, Milli-Q water, dried with a stream of nitrogen and immediately mounted into the SPR biosensor. First, the mixture of 12.5 mM NHS and 62.5 mM EDC (in Milli-Q water) was injected (10 minutes) to activate carboxylic groups. Then, Ab(cypD) at concentration of 10 μg/ml in SA10 was pumped through the flow cell until the response to the immobilized Ab(cypD) levelled off (~12 minutes). Then PBS_Na_ was applied (5 minutes) to remove the non-covalently attached Ab(cypD) from the surface. Finally, 500 mM EA was injected (5 minutes) to deactivate the unreacted carboxylic groups. The SA10 running buffer was exchanged for MES and then detection channels were exposed to 90 nM cypD in MES to reach surface saturation (~15 minutes), while the reference channels were kept in MES. Then, all the channels were switched to MES for at least 20 minutes and PBS_Na_ was applied (5 minutes) to remove the non-specifically bound cypD molecules (see Supplementary Fig. S1a for a typical example of the sensorgram for the immobilization of cypD). SPR chips used in the experiments in which 17β-HSD10, cypD and Aβ were incubated in solution, were treated in the same manner, except for the following differences. After the immobilization of Ab(cypD), PBS_Na_ was injected (1 minute) and 10 μg/ml BSA was pumped through the flow cell until the sensor response of 1.5 nm was reached. Subsequently, PBS_Na_ was injected (5 minutes) followed by 500 mM EA (5 minutes) (see Supplementary Fig. S1b for a typical example of the sensorgram for the immobilization of Ab(cypD)).

### Experimental: Study of interaction between 17β-HSD10 and cypD under variable environmental conditions

In this part of our study, cypD was immobilized on the surface of an SPR chip and the binding of 17β-HSD10 dissolved in different running buffers (containing different levels of ions or having different pH) was investigated. We used running buffers with: 1) varying concentrations of K^+^, Mg^2+^ and Ca^2+^ to study the effect of individual ions at pH of 7.4 and 7.8, and 2) constant concentration of one ion (K^+^ or Mg^2+^) and varying concentration of the other to study the effect of combined ions at pH of 7.4.

For all the experiments in this study, the running buffer was flowed along the functionalized SPR chip until the stable sensor baseline was reached. A solution of 500 nM 17β-HSD10 in the running buffer (V = 100 μl) was kept at 37 °C for 10 minutes after being freshly prepared to ensure a comparable degree of tetramerization of 17β-HSD10 in all the used samples. Then, the solution of 17β-HSD10 was diluted to 1:4 with the running buffer to obtain the final concentration of 100 nM and the sample was injected in both the detection (surface with immobilized cypD) and reference (surface without immobilized cypD) channels. The binding of 17β-HSD10 was observed for 7 minutes and then the running buffer was injected again. The surface of the SPR chip was then regenerated (up to five times) by injecting PBS_Na_ for 5 minutes. The final sensor response was determined as a difference between the responses of the detection and reference channels 3 minutes after switching to the running buffer. Each experiment was repeated at least three times on at least two independent SPR chips.

In the study of the effect of individual ions on the interaction between 17β-HSD10 and cypD, we used a running buffer composed of HEPES_1_ (10 mM; 200 μg/ml BSA, 5 mM Na^+^, pH 7.4), which was mixed with a constant volume of aqueous solutions of KCl, MgCl_2_ or CaCl_2_ at concentrations that ensured the same dilution of HEPES for all the studied concentrations of ions. The pH was adjusted by NaOH; NaCl was added to yield a concentration of 5 mM Na^+^. The concentration ranges used in our study were 0–140 mM for K^+^ and 0–2.5 mM for Mg^2+^ and Ca^2+^. A running buffer composed of HEPES_2_ (10 mM, 200 μg/ml BSA, 5 mM Na^+^, pH 7.8) mixed with particular concentrations of KCl and MgCl_2_ was used to study the effect of individual K^+^ and Mg^2+^ over the concentration ranges of 0–140 mM for K^+^ and 0–2.5 mM for Mg^2+^ at pH of 7.8.

In the study of the effect of combined ions on the interaction between 17β-HSD10 and cypD, a running buffer composed of HEPES_1_ was used to which a constant volume of the mixture of KCl and MgCl_2_ was added and concentrations of the ions were selected to obtain 15 mM K^+^ with 0–1 mM Mg^2+^, and 0.25 mM Mg^2+^ with 0–30 mM K^+^, respectively.

In addition, we compared the formation of 17β-HSD10/cypD complex at two different temperatures (25 °C and 37 °C) for three representative environmental conditions: 1) 15 mM K^+^, 0.1 mM Mg^2+^, 2) 15 mM K^+^, 1 mM Mg^2+^, and 3) 140 mM K^+^, 0.1 mM Mg^2+^ (all in HEPES_1_ running buffer). The temperature was adjusted to 37 °C by the temperature control unit of the SPR sensor prior to the injection of 17β-HSD10.

### Experimental: Study of interaction between 17β-HSD10 and cypD in the presence of Aβ

In this part of our study, two experimental formats were employed: (1) cypD was immobilized on the surface of an SPR chip and the binding of 17β-HSD10, Aβ_1–40_, Aβ_1–42_ or their mixtures was studied, and (2) samples containing cypD, 17β-HSD10, Aβ_1–40_, Aβ_1–42_ or their mixtures were incubated in solution, flowed over an SPR chip with immobilized Ab(cypD) and the binding of Ab(17β-HSD10) was measured to determine the amount of attached 17β-HSD10/cypD complex. The concentrations of the interacting molecules (cypD, 17β-HSD10, and Aβ) were chosen with respect to previously published *in vitro* studies^15,17,21^.

The first experimental format was employed in three different studies in which we investigated how the binding between 17β-HSD10 and cypD is affected by: (1) presence of Aβ_1–40_ and Aβ_1–42_, (2) excess of Aβ_1–40_, and (3) degree of oligomerization of Aβ_1–40_ and Aβ_1–42_. In all these studies, initially, HEPES_FINAL_ (10 mM HEPES, 200 μg/ml BSA, 5 mM Na^+^, 15 mM K^+^, 0.1 mM Mg^2+^, pH 7.4) was pumped across an SPR chip functionalized with cypD until the stable baseline was obtained. Then, the sample was injected into both the detection (surface with immobilized cypD) and the reference (surface without immobilized cypD) channels. The binding of 17β-HSD10 was monitored for 7 minutes (in the study of the effects of Aβ_1–40_ and Aβ_1–42_) or for 10 minutes (in the study of the effects of the excess of Aβ_1–40_ and degree of oligomerization of Aβ) and then HEPES_FINAL_ was injected again. The final (reference-compensated) sensor response was determined as the difference between the sensor responses obtained in the detection and reference channels 10 minutes after switching to the running buffer.

In the first study we investigated the effect of two different fragments of Aβ (Aβ_1–40_, Aβ_1–42_) on the interaction between 17β-HSD10 and cypD. Solutions of 17β-HSD10, Aβ_1–40_, Aβ_1–42_ and mixtures of these (see Supplementary Table S1 for details on the solutions used) were prepared in HEPES_FINAL_ (V = 100 μl) and incubated for 1 hour at 37 °C. After incubation, the solutions were diluted to 1:7.5 by HEPES_FINAL_ to obtain a final concentration of 100 nM of 17β-HSD10, 500 nM of Aβ_1–40_ and 500 nM of Aβ_1–42_, respectively. To evaluate the effect of excess of Aβ, different concentrations of Aβ (Aβ_1–40_ or Aβ_1–42_) were investigated, while the final concentration of 17β-HSD10 was held constant at 100 nM. A set of solutions of Aβ_1–40_ or Aβ_1–42_ at concentrations of 0.375, 0.75, 1.5, 3.75, 7.5 μΜ and a set of solutions of Aβ_1–40_ or Aβ_1–42_ at concentrations of 0.375, 0.5625 (for Aβ_1–42_ only), 0.75, 1.5, 3.75, 7.5 μΜ with 750 nM 17β-HSD10 in HEPES_FINAL_ (V = 100 μl) were prepared and incubated for 1 hour at 37 °C. After the incubation, the solution was further diluted by HEPES_FINAL_ to obtain final concentrations of 50, 75 (for Aβ_1–42_ only), 100, 200, 500 and 1000 nM of Aβ and 100 nM 17β-HSD10. Subsequently, to study the effect of oligomerization, binding experiments were performed for different oligomerization times for Aβ (Aβ_1–40_, Aβ_1–42_). A solution of Aβ_1–40_ (3.75 μΜ) and Aβ_1–42_ (3.75 μΜ) in HEPES_FINAL_ (V = 100 μl) was prepared and incubated for 0, 10, 30, 60 and 180 minutes at 37 °C. After the incubation, either 17β-HSD10 (15 μl, 0.5 mg/ml) or HEPES_FINAL_ (15 μl) was added to the solution of Aβ_1–42_ and HEPES_FINAL_ (15 μl) was added to the solution of Aβ_1–40_, and the solution was incubated for 10 more minutes. Then, the solution was diluted to 1:7.5 by HEPES_FINAL_ to obtain final concentrations of 100 nM and 500 nM, for 17β-HSD10 and Aβ, respectively. A freshly prepared 100 nM Aβ_1–40_ and 100 nM Aβ_1–42_ were included in the experiment for comparison.

In the second experimental format, we incubated 17β-HSD10, cypD, and Aβ (Aβ_1–40_ or Aβ_1–42_) together at a molar ratio of 1:5:10. This ratio was chosen so that the majority of cypD captured by Ab(cypD) is present in the form of a complex. The solutions of 17β-HSD10, cypD, Aβ_1–40_, Aβ_1–42_ and different mixtures of these (see Supplementary Table S2 for details on the solutions used) were prepared in HEPES_FINAL_ (V = 100 μl) and incubated for 3 hours at 37 °C. After the incubation, the samples were diluted to 1:7.5 by HEPES_FINAL_ to obtain the final concentrations of 250 nM, 50 nM and 500 nM for 17β-HSD10, cypD, and Aβ, respectively, and then flowed along the surface of an SPR chip with the immobilized Ab(cypD). After a 20-minute period during which the binding occurred, the sample was replaced with the running buffer. Finally, the 17β-HSD10/cypD complex was identified by measuring the response of the sensor to the injection of Ab(17β-HSD10) at a concentration of 10 μg/ml for 10 minutes.

## Supplementary information

Supplementary Information

## Data Availability

The data that support the findings of the current study are available from the corresponding author upon reasonable request.
