# Supplementary material for: In vitro study of interaction of 17β-hydroxysteroid dehydrogenase type 10 and cyclophilin D and its potential implications for Alzheimer’s disease
Source: Sci Rep. 2019 Nov 13;9:16700. doi: 10.1038/s41598-019-53157-7 (PMC6853915; doi:10.1038/s41598-019-53157-7)
Supplement: Supplementary file 1 — Supplementary Information [file 41598_2019_53157_MOESM1_ESM.pdf]

In vitro study of interaction of 17β-hydroxysteroid dehydrogenase type 10 and cyclophilin D and its potential implications for Alzheimer's disease

Erika Hemmerová[1], Tomáš Špringer[1], Zdenka Krištofiková[2], Jiří Homola[1*]

SUPPLEMENTARY INFORMATION

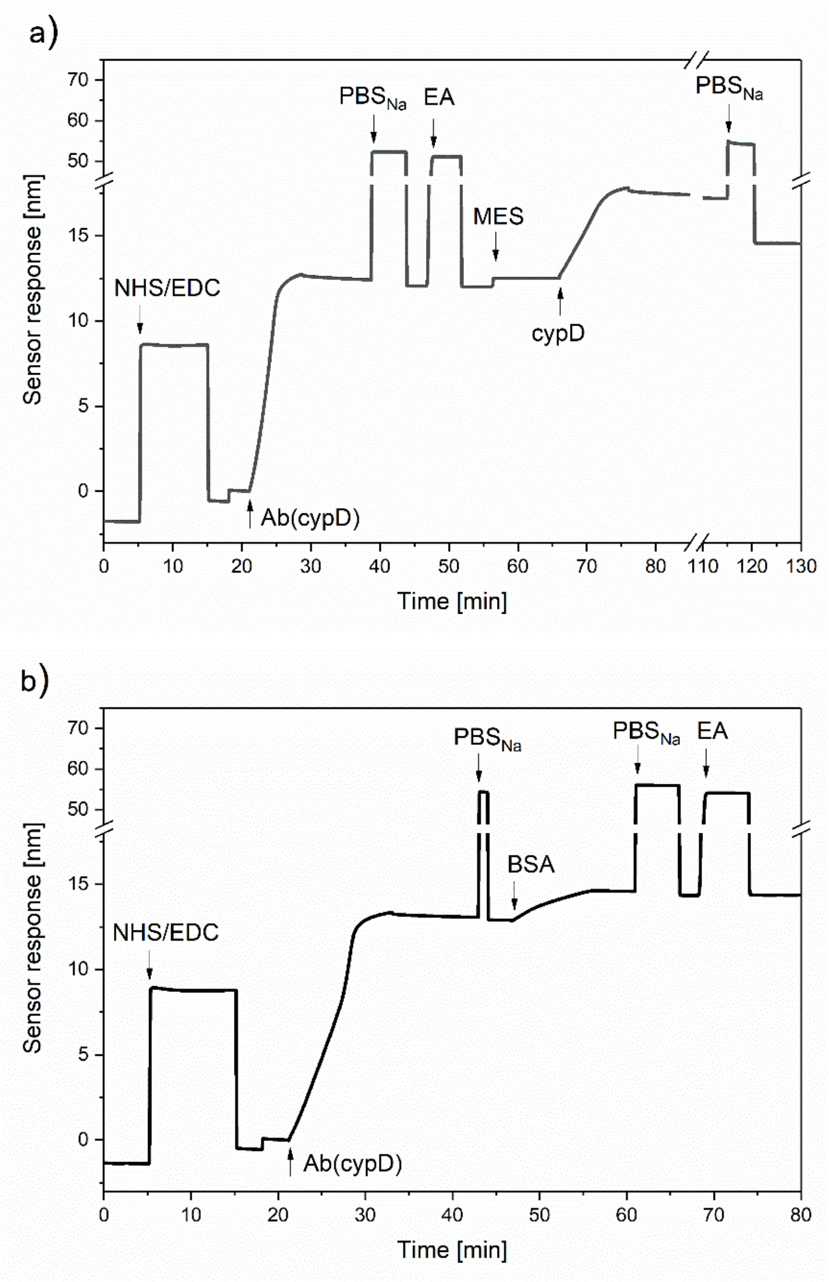

Supplementary Figure S1: Sensorgram of the functionalization of an SPR chip. a) immobilization of cypD via Ab(cypD) and b) immobilization of Ab(cypD).

Supplementary Table S1: Composition of the solutions used in the first experimental format (diluted Solutions 1-5 flowed over cypD immobilized on the surface of an SPR chip).

	17β-HSD10	$A\beta_{1-40}$	$A\beta_{1-42}$
Solution 1	0.750 μM	3.750 μM	x
Solution 2	0.750 μM	x	3.750 μM
Solution 3	0.750 μM	x	x
Solution 4	x	3.750 μM	x
Solution 5	x	x	3.750 μM

Supplementary Table S2: Composition of the solutions used in the second experimental format (diluted Solutions 1-12 flowed over Ab(cypD) immobilized on the surface of an SPR chip followed by the binding of Ab(17β-HSD10)).

	17β-HSD10	cypD	$A\beta_{1-40}$	$A\beta_{1-42}$
Solution 1	1.875 μM	0.375 μM	3.750 μM	x
Solution 2	1.875 μM	0.375 μM	x	3.750 μM
Solution 3	1.875 μM	0.375 μM	x	x
Solution 4	1.875 μM	x	3.750 μM	x
Solution 5	1.875 μM	x	x	3.750 μM
Solution 6	x	0.375 μM	3.750 μM	x
Solution 7	x	0.375 μM	x	3.750 μM
Solution 8	1.875 μM	x	x	x
Solution 9	x	0.375 μM	x	x
Solution 10	x	x	0.375 μM	x
Solution 11	x	x	x	3.750 μM
Solution 12	x	x	x	x

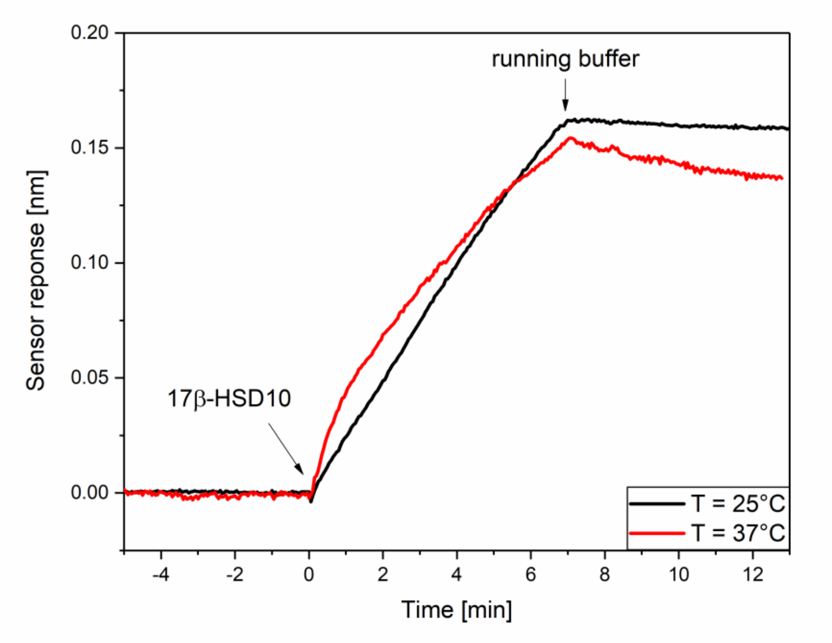

Supplementary Figure S2: Sensorgram corresponding to the binding of 17β-HSD10 to cypD at two different temperatures.

Supplementary Table S3: Comparison of sensor responses to the binding of 17β-HSD10 to cypD at the temperature of 25°C and 37°C for the selected ionic compositions of the running buffer.

Ionic composition of the running buffer	25°C	37°C
15 mM K^+, 0.1 mM Mg^{2+}	0.185 ± 0.028	0.155 ± 0.025
15 mM K^+, 1 mM Mg^{2+}	0.049 ± 0.025	0.032 ± 0.020
140 mM K^+, 0.1 mM Mg^{2+}	0.097 ± 0.015	0.101 ± 0.004

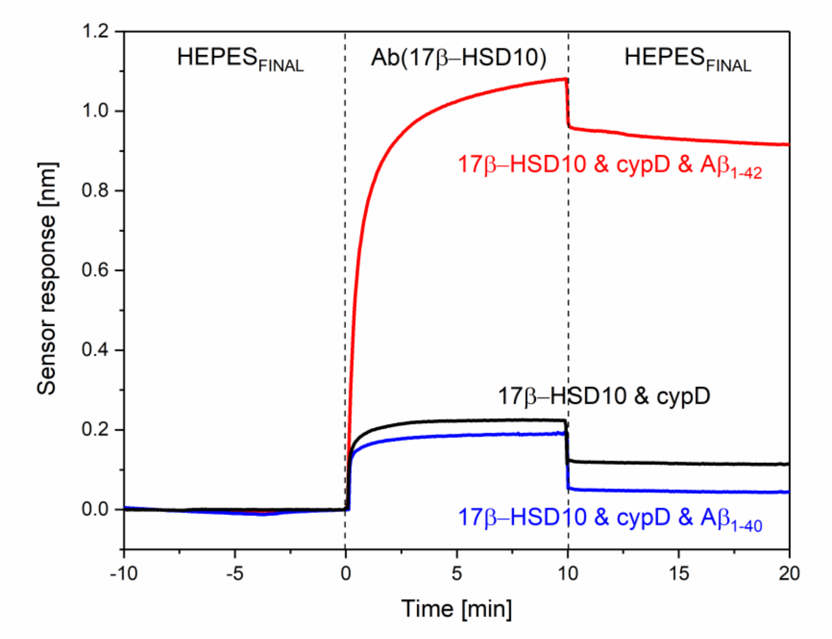

Supplementary Figure S3: Sensorgram corresponding to the binding of Ab(17β-HSD10) to a surface previously exposed to the mixture of 17β-HSD10 and cypD, or 17β-HSD10 and cypD and Aβ$_{1-40}$/Aβ$_{1-42}$ (diluted Solutions 1-3 in Supplementary Table S2).

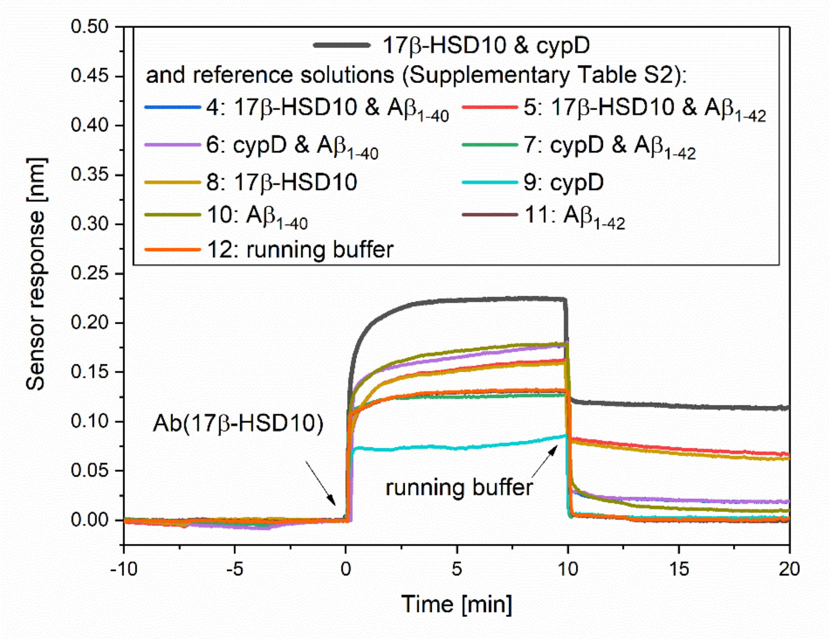

Supplementary Figure S4: Sensorgram corresponding to the binding of Ab(17β-HSD10) to a surface previously exposed to the mixture of 17β-HSD10 and cypD and reference solutions (consisting of different combinations of 17β-HSD10, cypD and Aβ$_{1-40}$/Aβ$_{1-42}$; diluted Solutions 4-12 in Supplementary Table S2).

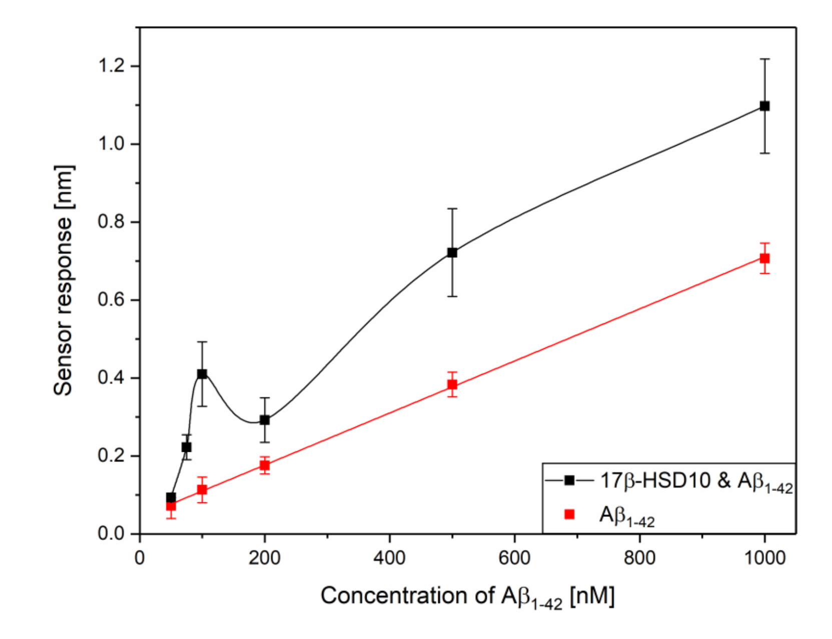

Supplementary Figure S5: Dependence of the binding of A$\beta_{1\text{-}42}$ and A$\beta_{1\text{-}42}$ incubated with17β-HSD10 to the immobilized cypD on concentration of A$\beta_{1\text{-}42}$.
